# Glutathione peroxidase 4 as an emerging therapeutic target in osteoarthritis: focus on ferroptosis

**DOI:** 10.3389/fcell.2025.1737456

**Published:** 2026-01-12

**Authors:** Lin Zhang, Jinglin Li, Xuxu Yang, Lidan Yang

**Affiliations:** Department of Orthopedics, Affiliated Hospital of Zunyi Medical University, Zunyi, China

**Keywords:** chondrocyte, extracellular matrix, ferroptosis, GPX4, osteoarthritis

## Abstract

Osteoarthritis (OA) is a degenerative joint disease characterized by articular cartilage degradation, extracellular matrix breakdown, low-grade chronic inflammation, and pain. Its etiology is complex and treatment options are limited. In recent years, ferroptosis, a regulated form of cell death driven by iron-dependent lipid peroxidation, has gained significant attention in OA pathogenesis. Glutathione peroxidase 4(GPX4), serves as the central enzyme that halts lipid peroxidation and inhibits ferroptosis. Its expression and activity are altered in OA cartilage under pathological conditions, suggesting a crucial role for GPX4 in OA pathogenesis and treatment. This review summarizes the molecular characteristics and antioxidant functions of GPX4, evaluates experimental evidence linking GPX4 and ferroptosis in OA, outlines upstream and downstream molecular mechanisms regulating GPX4, and summarizes therapeutic strategies targeting GPX4, including pharmacological, gene, and combination therapies. It also discusses current research challenges and future directions. Finally, key pathways and strategic recommendations for translating GPX4 and ferroptosis research into clinical OA treatments are proposed.

## 1 Introduction

Osteoarthritis (OA), is an extremely prevalent degenerative joint disease worldwide (GBD 2021 Osteoarthritis Collaborators, 2023). Its primary clinical manifestations include joint pain, stiffness, and limited mobility. Pathological features encompass degenerative changes in articular cartilage, alterations in subchondral bone structure, low-grade chronic inflammation of the synovium, and overall joint dysfunction (Hunter and Bierma-Zeinstra, 2019). The pathogenesis of OA is highly complex, involving interactions among multiple factors such as abnormal mechanical loading, age-related metabolic changes, persistent inflammatory responses, dysregulated extracellular matrix(ECM) metabolism, and genetic susceptibility. These factors collectively contribute to the progressive decline in chondrocyte function and accelerated breakdown and loss of ECM(2). In clinical practice, while physical therapy, analgesic medications, and even joint replacement surgery can alleviate symptoms, no currently available drug therapies can effectively halt or reverse cartilage degeneration (Cho et al., 2021; Li S. et al., 2023). Therefore, exploring novel molecular mechanisms underlying OA development and identifying viable intervention targets are of paramount scientific and clinical importance.

In recent years, ferroptosis, a regulated form of cell death driven by iron-dependent lipid peroxidation, has been identified and rapidly become a frontier in cell death research. Since its systematic conceptualization by Dixon et al. (2012) in 2012 as an iron-dependent, non-apoptotic form of programmed cell death, its molecular mechanisms and biological significance in physiological and pathological states have been progressively elucidated. Glutathione peroxidase 4(GPX4), is recognized as a key effector molecule in defending against ferroptosis. This enzyme specifically catalyzes the reduction of lipid peroxides within cell membranes to their corresponding non-toxic lipid alcohols, thereby effectively blocking the propagation of lipid peroxidation chain reactions. Further studies have shown that genetic knockout of GPX4 induces cell death with characteristic features of ferroptosis, genetically confirming its central role in maintaining cellular lipid homeostasis and defending against oxidative damage (Dixon et al., 2012; Friedmann Angeli et al., 2014; Liu J. et al., 2024).

In OA research, numerous basic and translational studies have recently demonstrated that chondrocytes, under various pathological stimuli such as iron overload, inflammatory cytokine exposure, and mechanical overload, exhibit typical molecular and morphological hallmarks of lipid peroxidation accumulation and ferroptosis. Concurrently, downregulation or functional loss of GPX4 has been closely associated with OA pathological progression. Multiple lines of evidence from *in vitro* and *in vivo* studies indicate significant iron accumulation and elevated lipid peroxidation levels in OA-affected tissues. Furthermore, using specific ferroptosis inhibitors such as ferrostatin-1 or interventions that enhance GPX4 activity can, to some extent, restore chondrocyte viability and reduce ECM degradation (Yao et al., 2021; Xu Y. et al., 2023; Pang et al., 2024). Additionally, research has revealed that mechanical overload can induce GPX4-regulated ferroptosis by activating the Piezo1 ion channel, mediating calcium influx (Wang S. et al., 2022). This finding suggests crosstalk between mechanical stress, *via* specific ion channels, and intracellular oxidative stress pathways, collectively influencing chondrocyte fate.

Given the central role of GPX4 in inhibiting lipid peroxidation, preventing ferroptosis, and maintaining the structural integrity of cellular and mitochondrial membranes (Stockwell et al., 2017; Miao et al., 2022; Xie et al., 2025; Wang Q. et al., 2024), a systematic and in-depth review of GPX4’s expression changes, regulatory networks, and association with ferroptosis in OA, as well as its potential as a therapeutic target, holds significant theoretical value and clinical translational relevance. While previous reviews have focused on the overall role of ferroptosis in OA or orthopedic diseases, they often lack an in-depth analysis centered on GPX4 as the core inhibitory factor. This review aims to synthesize current research around GPX4. It will first elaborate on the basic concept of ferroptosis and its related experimental evidence in OA. It will then systematically discuss the upstream regulatory mechanisms of GPX4 in the context of OA, including the System Xc^−^ and nuclear factor erythroid 2-related factor 2(Nrf2) signaling pathways, as well as its downstream biological effects, such as impacts on chondrocyte survival, inflammatory responses, and ECM metabolism. Finally, the review will discuss therapeutic strategies targeting GPX4 and ferroptosis, including pharmacological and gene therapies, and analyze the main challenges in this field and potential future research directions.

## 2 GPX4 and ferroptosis in OA

### 2.1 Definition, biological characteristics of ferroptosis and Its distinction from other cell death forms

Ferroptosis is an iron-dependent form of regulated cell death primarily triggered by the aberrant accumulation of lipid peroxides. Recently, the role of this cell death modality in the pathogenesis of various diseases has garnered increasing attention (Čepelak et al., 2020). Ferroptosis exhibits fundamental differences from classical cell death forms such as apoptosis, necroptosis, autophagy, and necrosis at the levels of biochemical pathways, morphological changes, and genetic regulation, establishing it as a distinct cell death category. The biological characteristics of ferroptosis are primarily reflected in several key aspects. First, its occurrence strictly depends on intracellular iron, which catalyzes the Fenton reaction, efficiently promoting the generation of reactive oxygen species (ROS) that directly drive the peroxidation of polyunsaturated fatty acids(PUFAs) in cell membranes. Notably, disruption of intracellular iron homeostasis, particularly pathological accumulation of free iron, significantly enhances cellular susceptibility to ferroptosis. Second, the execution core of ferroptosis involves the peroxidation of polyunsaturated fatty acid-containing phospholipids(PUFA-PLs) in membrane lipids, leading to the massive accumulation of cytotoxic lipid peroxidation derivatives such as specific lipid hydroperoxides and reactive aldehydes like 4-hydroxynonenal(4-HNE), ultimately causing irreversible damage to plasma membrane integrity (Guo et al., 2024). Morphologically, the most typical alterations in ferroptotic cells are concentrated in mitochondria, characterized by a marked reduction in mitochondrial volume, increased membrane density, and reduction or disappearance of mitochondrial cristae, while the nucleus remains relatively intact, lacking apoptotic features like chromatin condensation or nuclear membrane rupture (Wang et al., 2020). This unique morphological profile clearly distinguishes it from apoptosis and necrosis. Furthermore, the ferroptotic process can be precisely modulated by specific small molecules. For instance, erastin can induce ferroptosis by inhibiting system Xc^−^ activity, depleting intracellular reduced glutathione(GSH), whereas RSL3 directly covalently binds to and inhibits GPX4 activity (Wang L. et al., 2022). Conversely, radical-trapping antioxidants like ferrostatin-1 and liproxstatin-1 effectively neutralize lipid peroxyl radicals, blocking the chain reaction and specifically inhibiting ferroptosis. These unique biological characteristics not only clearly differentiate ferroptosis from other known cell death forms but also strongly suggest its potential key pathogenic role in various chronic diseases including OA, positioning it as a promising drug intervention target (Dixon et al., 2012; Stockwell et al., 2017).

### 2.2 The central role of GPX4 in ferroptosis

GPX4, a selenocysteine-dependent antioxidant enzyme, occupies an irreplaceable central position in the cellular defense network against ferroptosis. Its core biochemical function is to utilize reduced GSH as an essential cofactor to specifically catalyze the reduction of lipid hydroperoxides within membrane phospholipids to their corresponding harmless lipid alcohols. This reaction directly interrupts the propagation chain of lipid peroxidation, thereby preventing the execution of ferroptosis at the molecular level. The central role of GPX4 in ferroptosis can be elaborated from multiple dimensions. Its mechanism of action exhibits high substrate specificity. Unlike other glutathione peroxidase family members, GPX4 displays a unique catalytic preference for peroxides embedded in biomembrane phospholipids, enabling it to directly eliminate the most destructive lipid peroxidation products located on cellular and organellar membranes, safeguarding membrane stability and function at the source. GPX4 enzymatic activity strictly depends on a continuous and sufficient supply of intracellular GSH. *De novo* GSH synthesis is tightly regulated by the cystine/glutamate antiporter system Xc^−^, whose core subunit is Solute carrier family 7 member 11 (SLC7A11). Therefore, the System Xc^−^–GSH–GPX4 axis constitutes a complete and finely tuned antioxidant defense chain (Guo et al., 2024). Genetical evidence from key studies confirms that eliminating GPX4 expression *via* genetic knockout in various cell types, or directly targeting its active site with small molecule inhibitors like RSL3, consistently and efficiently induces typical ferroptosis (Wang L. et al., 2022). At the whole-organism level, global or tissue-specific knockout of GPX4 leads to acute organ failure and animal death. These robust *in vitro* and *in vivo* evidences collectively establish the indispensable physiological protective role of GPX4 in maintaining organismal redox homeostasis and defending against ferroptosis (Friedmann Angeli et al., 2014). Although GPX4 is the primary molecule inhibiting ferroptosis, recent research has uncovered other parallel compensatory defense mechanisms within cells, reflecting the redundancy of biological systems. For example, Ferroptosis Suppressor Protein 1(FSP1), localized to the plasma membrane, exerts antioxidant functions by mediating the regeneration of coenzyme Q10(CoQ10). Dihydroorotate dehydrogenase (DHODH) within mitochondria is believed to help maintain redox balance in this organelle (Bersuker et al., 2019). The existence of these parallel pathways suggests a multi-layered and complementary nature of cellular mechanisms against ferroptosis. However, in most physiological and pathological contexts, GPX4 is still widely recognized as the primary and most central effector molecule within this defense network. Consequently, the defense axis constituted by GPX4 and GSH is generally considered the core molecular apparatus for cellular resistance to ferroptosis, making it a focal point in OA pathogenesis research and targeted therapy exploration.

### 2.3 Experimental evidence for ferroptosis in OA

In recent years, as our understanding of ferroptosis has deepened, numerous basic and preclinical studies have accumulated compelling evidence supporting its active role in OA pathology. These studies, conducted using both *in vitro* cultured chondrocytes and *in vivo* OA animal models, have provided strong molecular and phenotypic data. Together, they systematically construct a complete logical chain from disease etiology to potential intervention. At the joint microenvironment level, analyses of synovial fluid and cartilage tissue samples from OA patients, as well as studies in surgically induced such as the DMM model or chemically induced animal OA models, consistently observe clear iron metabolism abnormalities and significant iron accumulation locally in affected joints. This pathological iron overload provides a continuous substrate for the Fenton reaction, constituting a fundamental and persistent risk factor driving lipid peroxidation and inducing ferroptosis (Zhang Y. et al., 2025; Cao et al., 2023). Regarding the expression of core defense molecules, multiple independent research teams have consistently reported significantly downregulated GPX4 protein and mRNA levels in OA patient cartilage tissues, or in vitro chondrocytes treated with inflammatory cytokines like interleukin-1β(IL-1β) to mimic the OA inflammatory environment, or under direct iron overload conditions. This is accompanied by depletion of intracellular GSH levels. Conversely, markers of lipid peroxidation, such as malondialdehyde(MDA) and 4-HNE, are significantly increased. Notably, the worsening of these oxidative damage indicators often correlates temporally and in degree with the upregulated expression of key ECM-degrading enzymes such as MMP13 and ADAMTS5, strongly suggesting an intrinsic, potentially causal, molecular link between ferroptosis and cartilage matrix destruction (Yao et al., 2021; Miao et al., 2022). At the level of functional intervention and therapeutic validation, multiple animal studies provide the most direct evidence. Specifically, intra-articular injection or systemic administration of specific ferroptosis inhibitors such as ferrostatin-1, or pharmacologically for example, using natural products or genetically for example, using overexpression vectors upregulating GPX4 expression, activating SLC7A11, or the key transcription factor Nrf2, can, to varying degrees, restore cartilage morphology, reduce chondrocyte death, and significantly lower the expression levels of ECM degradation markers in OA animal models. The success of these interventions strongly demonstrates that ferroptosis plays an active, pathogenic role in OA pathogenesis and that this process is, to some extent, reversible by specific pharmacological strategies (Wang J. et al., 2024; Wu et al., 2025a; Dong et al., 2025). Regarding mechanism linkage and integration, research further reveals that the two most common OA pathogenic stimuli—chronic inflammatory cytokines such as IL-1β and abnormal mechanical stress—can effectively induce ferroptosis in chondrocytes. Particularly noteworthy, mechanical overload can activate the mechanosensitive ion channel Piezo1, mediating calcium influx, which subsequently triggers the downstream GPX4-regulated ferroptosis pathway (Yao et al., 2021; Wang S. et al., 2022). This establishes a direct and important mechanistic bridge between the classic mechanical pathogenic factor in OA and the emerging oxidative stress-mediated cell death pathway. This multi-level, multi-angle evidence chain—from clinical observation to cell models and animal interventions—collectively supports ferroptosis as a critical link in OA cartilage pathology, with GPX4 dysfunction permeating this process. Systematic reviews and numerous original studies have tightly integrated GPX4 into the complex pathogenic network of OA and clearly pointed out the potential and significant therapeutic value of targeting the GPX4 and ferroptosis axis.

### 2.4 Correlation between GPX4 downregulation and OA severity

Extensive clinical pathological analyses and rigorously controlled animal model studies collectively reveal a clear pattern: the protein expression level or biological activity of GPX4 typically shows a significant negative correlation with the severity of OA lesions. This correlation provides the most direct evidence for the core protective role of GPX4 in OA. Specifically, *in vitro* studies, most cell models focus on articular chondrocytes, confirming that GPX4 expression is lower in model groups compared to normal groups. A very limited number of studies have also focused on synoviocytes and synovial fibroblast-like synoviocytes(FLS) models treated with LPS or IL-1β. For example, Luo et al. found decreased GPX4 levels in LPS-treated synoviocytes, while Icariin could inhibit synoviocyte ferroptosis and enhance cell survival in lipopolysaccharide-induced synoviocytes *via* the Xc^−^/GPX4 axis (Luo and Zhang, 2021). Similarly, Hu et al. found that Lipoxin A4 could increase GPX4 levels by activating the ESR2/LPAR3/Nrf2 axis, inhibit FLS ferroptosis, thereby reducing synovial inflammation, alleviating cartilage degeneration, and mitigating pain and pathological progression in knee OA (Hu et al., 2024). In human specimens and OA models established *via* surgery such as the DMM model or ACLT, immunohistochemical or Western blot analyses of OA cartilage and synovial tissues show significantly lower GPX4 expression in lesioned areas compared to adjacent relatively normal tissues or normal control mice and patients. The degree of this downregulation correlates positively with the severity of cartilage degeneration, being most pronounced in advanced lesions characterized by surface fibrillation and severe proteoglycan loss (Miao et al., 2022; Lv Z. et al., 2022; Ma et al., 2024). Conversely, cartilage-specific overexpression of GPX4 *via* transgenic technology, or restoration of its activity using small molecule drugs such as certain natural compounds or ferroptosis inhibitors, can significantly delay cartilage degeneration, improve subchondral bone sclerosis and osteophyte formation, and alleviate synovitis in model animals (Xiao et al., 2024), which directly confirms the protective role of GPX4 in OA progression from a gain-of-function perspective. Beyond transcriptional regulation by factors like Nrf2, GPX4 protein abundance and stability are finely regulated by complex post-translational modifications and protein interaction networks. For instance, recent studies indicate that GPX4 stability is regulated by the ubiquitin-proteasome pathway, where its protein may be recognized and degraded by specific E3 ubiquitin ligases. Molecules like the cell cycle protein p21 have been reported to interact directly with GPX4, competitively or allosterically affecting its ubiquitination rate, thereby stabilizing GPX4 and enhancing cellular resistance under oxidative stress (Zheng et al., 2024). These findings provide new molecular basis for explaining the heterogeneity and differential progression observed in OA across different disease stages, affected joints, and individuals. Integrating these research results, GPX4 can be regarded not only as a potential biomarker sensitively reflecting OA pathological severity and disease stage but also as a therapeutically promising target with core protective functions in OA progression.

## 3 Molecular mechanisms of GPX4 in OA

### 3.1 Upstream regulation of GPX4: System Xc^−^–GSH axis and Nrf2 pathway

#### 3.1.1 Critical role of System Xc^−^ and GSH supply

**FIGURE 1:**
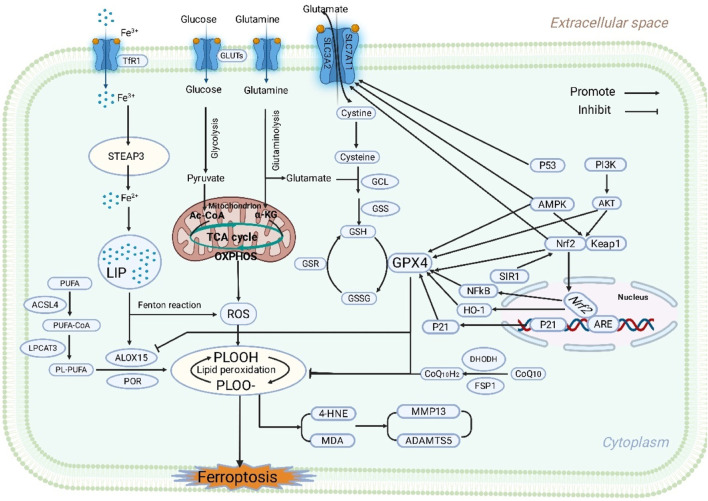
Molecular mechanisms of GPX4 in chondrocyte of OA.

System Xc^−^ is a heterodimeric membrane transport protein composed of a catalytic subunit, SLC7A11, and a regulatory subunit, SLC3A2, linked by a disulfide bridge. Its function is to perform cystine/glutamate exchange across the plasma membrane. This transport activity is crucial for maintaining intracellular synthesis of reduced GSH, as the imported cystine serves as the rate-limiting precursor for GSH production. GSH, acting as an indispensable electron donor in the GPX4 enzymatic reaction, directly determines the enzymatic activity of GPX4 in reducing lipid peroxides (Huo et al., 2024). Consequently, any factor inhibiting SLC7A11 function or downregulating its expression leads to intracellular GSH depletion, thereby depriving GPX4 of its essential substrate and ultimately triggering irreversible ferroptosis. In the pathological environment of OA, various pathogenic stimuli have been confirmed to significantly impair System Xc^−^ function. For example, sustained stimulation by inflammatory cytokines such as IL-1β and TNF-α, persistent oxidative stress, and pathological iron overload states have all been shown to significantly suppress SLC7A11 expression or its transport activity (Xiao et al., 2024; Ruan et al., 2024; Zeng et al., 2024; He R. et al., 2024). This inhibition renders GPX4 unable to perform its protective function due to shortage of its required co-substrate GSH, making chondrocytes more susceptible to ferroptosis. The molecular axis constituted by System Xc^−^–GSH–GPX4 and its dysregulation in OA have been repeatedly verified and confirmed in multiple independent studies, establishing it as a closely watched potential therapeutic target for intervening in ferroptosis (Figure 1). However, it is noteworthy that current evidence supporting this mechanism primarily comes from *in vitro* chondrocyte models or rodent OA animal models, with relatively limited direct validation in human cartilage tissue. Particularly, the significant variation in inducing conditions such as IL-1β, oxidative stress, and iron load across different studies makes it unclear whether SLC7A11 downregulation is a universal mechanism in OA, lacking systematic assessment of consistency across models.

#### 3.1.2 Transcriptional regulation of GPX4 by the Nrf2 pathway

Nuclear factor erythroid 2-related factor 2(Nrf2), is a master transcriptional regulator of the cellular response to oxidative stress. Under physiological conditions, Nrf2 is bound to its cytoplasmic inhibitor, Kelch-like ECH-associated protein 1(Keap1), and remains inactive. Upon cellular challenge by electrophiles or ROS, Nrf2 is activated and translocates to the nucleus, where it initiates the transcription of a battery of target genes containing antioxidant response elements or ARE. These target genes encompass a wide range of antioxidant enzymes and GSH synthesis-related genes, including the key System Xc^−^ component SLC7A11. Activating Nrf2 can directly or indirectly transcriptionally upregulate GPX4 expression and cooperatively induce other antioxidant molecules, forming a comprehensive defense network against lipid peroxidation. In OA research, recent evidence indicates that pharmacological activation of the Nrf2–GPX4 signaling axis using bioactive natural products or synthetic small molecule agonists can effectively mitigate ferroptosis in vitro cultured chondrocytes and *in vivo* OA animal models, while significantly suppressing ECM degradation progression (Wu et al., 2025b; Zhang Z. et al., 2025; He Q. et al., 2023; Ruan et al., 2023). This makes targeting the Nrf2–GPX4 pathway a highly promising and important direction for developing OA disease-modifying therapies (Figure 1). However, it must be emphasized that current research on the Nrf2–GPX4 axis remains dominated by experiments pharmacologically activating Nrf2. The specificity and effective *in vivo* concentrations of many natural products or small molecule agonists are debatable, with some effects possibly stemming from their broad-spectrum antioxidant capacity rather than genuine Nrf2-specific action. Furthermore, existing studies have not adequately distinguished the relative contributions of transcriptional upregulation of GPX4 *versus* increased protein stability to the outcomes, leaving some mechanistic explanations still at the inferential level. Additionally, inconsistent findings regarding the role of Nrf2 at different stages of OA suggest that Nrf2 may have stage-dependent or even biphasic effects. Therefore, although current data support Nrf2 as an important regulatory point upstream of GPX4, its precise mode of action at various stages of OA progression and the long-term safety of pharmacological activation require further systematic investigation.

### 3.2 Post-translational modifications and protein stability of GPX4

The protein abundance and functional activity of GPX4 are not solely determined by transcriptional levels but are precisely regulated by a series of complex post-transcriptional and post-translational modification events, including but not limited to ubiquitination, interaction with specific proteins, and potential phosphorylation and glycosylation (Huo et al., 2024). Recent research found that the cell cycle protein p21 can influence GPX4 stability through direct protein-protein interaction. Under oxidative stress, this interaction helps maintain GPX4 protein levels, thereby exerting cytoprotective effects. Functional experiments confirmed that knocking down p21 expression in a DMM surgery-induced mouse OA model exacerbated articular cartilage degradation and was accompanied by a significant decrease in GPX4 protein levels *in vivo*, demonstrating that p21 plays an important anti-ferroptotic role in OA progression by modulating GPX4 stability (Zheng et al., 2024). It should be noted that the evidence supporting this mechanism mainly originates from a single research group, and its reproducibility across different OA models has not been fully validated. Therefore, whether the p21–GPX4 axis is a universally present protective mechanism in OA still awaits broader data support (Figure 1). Furthermore, under OA-related inflammatory and metabolic stress environments, cells may promote GPX4 ubiquitination and subsequent proteasomal degradation by inducing specific E3 ubiquitin ligase activity, thereby increasing cellular susceptibility to ferroptosis (Su et al., 2025; Wang K. et al., 2025; Lu et al., 2025). Regarding the ubiquitination regulation of GPX4, current evidence remains more speculative. Although analyses suggest that inflammation and oxidative stress promote E3 ubiquitin ligase activity, the specific E3 ligase responsible for GPX4 ubiquitination, its target sites, and their actual changes in human OA tissues have not been clearly identified. This indicates that research on GPX4 protein stability regulation is still in its early stages, with incomplete mechanistic chains. In summary, post-translational regulation of GPX4 provides new therapeutic entry points for OA intervention, but the strength of evidence, reproducibility, and clinical relevance of this research still need significant enhancement. Future studies require more validation using gene knockout models and human-derived samples.

### 3.3 Downstream effects of GPX4

#### 3.3.1 Inhibition of lipid peroxidation and maintenance of membrane and mitochondrial integrity

The most direct and core downstream biological effect of GPX4 lies in its ability to specifically catalyze the reduction of lipid peroxides on cellular and organellar membranes to harmless lipid alcohols. This biochemical reaction directly prevents the rupture of biomembrane structures and the abnormal accumulation of toxic lipid-derived signaling molecules, thereby robustly maintaining the structural integrity and normal physiological function of plasma and mitochondrial membranes. Mitochondria, as the cell’s powerhouses and crucial signaling hubs, suffer severe consequences from membrane lipid peroxidation, including significant disruption of energy metabolism and decisive alteration of cell fate through amplification of apoptotic or other death signaling pathways. The presence and proper function of GPX4 effectively block this vicious cycle initiated by lipid peroxidation at the molecular level, representing a key factor for maintaining chondrocyte survival under OA pathological stress (Yao et al., 2021; Dong et al., 2025) (Figure 1). However, it should be pointed out that the methods used to determine “mitochondrial ferroptosis” in existing studies are relatively scattered, involving membrane potential, ROS, ultrastructural changes, among others, many of which are not specific to ferroptosis. Therefore, whether GPX4 deficiency-induced mitochondrial damage is the “cause” or an “accompanying phenomenon” of ferroptosis remains somewhat controversial in OA research.

#### 3.3.2 Reduction of ECM degrading enzyme induction and inflammatory cascades

Reactive aldehydes such as 4-HNE and MDA and other secondary products generated during lipid peroxidation are not merely markers of cellular damage but also potent biological signaling molecules. These substances can activate key pro-inflammatory signaling pathways such as nuclear factor kappa B (NF-κB), thereby inducing the gene transcription and protein secretion of extracellular matrix (ECM) degrading enzymes like matrix metalloproteinase 13 (MMP13) and A Disintegrin and Metalloproteinase with Thrombospondin Motifs 5 (ADAMTS5) (Miao et al., 2022; Zhang et al., 2023; Jiang et al., 2021). The upregulation of these enzymes directly leads to excessive breakdown of core ECM components in cartilage, such as type II collagen and aggrecan. By its upstream action of inhibiting lipid peroxidation, GPX4 can indirectly but effectively reduce the expression levels of these destructive enzymes, thereby delaying or preventing ECM breakdown (El-Bikai et al., 2010). Animal model studies consistently show that pharmacological inhibition of ferroptosis or genetic upregulation of GPX4 expression leads to significantly reduced MMP and ADAMTS expression in cartilage tissue, accompanied by marked improvement in cartilage structural integrity (Dong et al., 2025; Xiao et al., 2024) (Figure 1).

#### 3.3.3 Impact of GPX4 on immune-synovial interactions and joint microenvironment

The contribution of GPX4 and ferroptosis to OA pathology extends far beyond direct protection of chondrocytes. They also profoundly influence the behavior of other cells within the joint, particularly the activation state of immune cells like FLS and infiltrating macrophages, by modulating signals such as lipid peroxidation products and debris released from dying cells. This participation contributes to the amplification and persistence of low-grade chronic inflammation within the joint cavity (Hu et al., 2024). Specifically, lipid peroxidation products such as 4-HNE can act as endogenous danger signals, activating the NF-κB pathway and the NOD-like receptor family pyrin domain containing 3 (NLRP3) inflammasome, subsequently triggering the release of potent inflammatory cytokines like IL-1β and tumor necrosis factor-alpha (TNF-α). These cytokines not only directly damage chondrocytes but also act back on the synovium and other chondrocytes, creating a self-amplifying vicious cycle of inflammation along the “cartilage-synovium” axis. This drives more chondrocytes into oxidative damage and ferroptosis, thereby exacerbating the overall pathological progression of OA (Grigolo et al., 2003; Chen B. Y. et al., 2024). Supporting this view are clinical and preclinical evidences, including the detection of significantly elevated 4-HNE levels in OA patient synovial fluid compared to normal (Morquette et al., 2006); significantly enhanced fluorescence signals for MDA and 4-HNE measured in OA patient cartilage tissue by techniques like immunofluorescence staining (Shah et al., 2005); and elevated 4-HNE levels found in both cartilage and synovial fluid of surgically induced canine OA models, where direct intra-articular injection of 4-HNE could replicate the cartilage destruction phenotype, accompanied by significant upregulation of MMP13, ADAMTS5, and cyclooxygenase-2 (COX2) expression (Shi et al., 2014). Multiple studies indicate that inhibiting ferroptosis not only directly protects chondrocytes but also effectively reduces synovial tissue inflammation indicators and the overall inflammatory level within the joint cavity, suggesting that the protective effect of GPX4 has systemic significance across different tissue types. However, current evidence is mostly from observational or single-model experiments, lacking systematic cross-tissue validation. For instance, elevated 4-HNE in synovial fluid has been reported in multiple studies, but significant variations in detection methods and patient stratification such as disease stage and inflammation degree between studies mean the strength of evidence for 4-HNE as a driver of OA inflammation amplification still requires cautious interpretation.

Furthermore, the complex intercellular communication network within the joint, involving exosomes and various cytokines secreted by synovial cells or chondrocytes themselves, also participates in the fine-tuning of ferroptosis susceptibility. They modulate the expression of key molecules like SLC7A11 and GPX4, or deliver specific microRNAs (miRNAs) and long non-coding RNAs (lncRNAs) to alter the fate of target cells. For example, exosomes derived from OA patient FLS can carry and deliver miR-19b-3p, which exacerbates ferroptosis and injury in recipient chondrocytes by targeting and inhibiting SLC7A11 expression (Kong et al., 2023); conversely, transfection with a miR-181b inhibitor was confirmed to inhibit chondrocyte ferroptosis by increasing SLC7A11 and GPX4 expression levels (Wang D. et al., 2023). On the other hand, exosomes from cells with reparative potential, such as bone marrow mesenchymal stem cells (MSCs) or amniotic epithelial cells, have been found to downregulate the pro-ferroptotic lipid metabolism enzyme ACSL4, or upregulate parallel anti-ferroptosis pathways like FSP1/GPX4, thereby attenuating ferroptosis and promoting cartilage repair (Wang Y. et al., 2024). Thus, the regulation of GPX4 is not merely an isolated event within individual chondrocytes but a systemic biological process involving complex network interactions among multiple cell types throughout the joint microenvironment *via* paracrine and other secretory pathways. However, current research is mostly based on *in vitro* validation, and its stability, targeting, and potential disease stage dependence in the real joint microenvironment remain unclear. Overall, although potential interactions exist between GPX4 and synovial inflammation, this field is still in its early stages and requires more *in vivo* data support.

### 3.4 Synergistic interactions of GPX4 with ACSL4, LPCAT3, and others

The occurrence of ferroptosis strongly depends on the presence of its specific substrates—PUFAs within membrane phospholipids. Acyl-CoA synthetase long-chain family member 4 (ACSL4) is a key enzyme in lipid metabolism responsible for catalyzing the conjugation of free PUFAs with coenzyme A. The activated acyl-CoAs can then be incorporated into membrane phospholipids. Therefore, ACSL4 is a key promoter determining the content of oxidizable PUFAs in membrane phospholipids, thereby facilitating ferroptosis (Chen X. et al., 2021). In OA research, multiple independent studies have found that ACSL4 expression is upregulated in degenerated cartilage tissue. Its increased expression significantly elevates the pool of oxidizable lipid substrates in cell membranes, thereby comprehensively increasing chondrocyte susceptibility to ferroptosis. Conversely, inhibiting ACSL4 expression or activity through drugs, specific lncRNAs, or other interventions has been proven to effectively reduce lipid peroxidation levels and exert protective effects on chondrocytes (Liu J. et al., 2024; Cao S. et al., 2025; Guan et al., 2025; Zhou et al., 2023). Lysophosphatidylcholine acyltransferase 3 (LPCAT3) works synergistically with ACSL4, responsible for incorporating the PUFA-CoA activated by ACSL4 into membrane phospholipids, completing membrane remodeling. Therefore, coordinately regulating the expression and activity of lipid metabolism enzymes like ACSL4 and LPCAT3 is considered an important intervention strategy from the perspective of reducing the source of ferroptosis substrates (Habaxi et al., 2024). Recently, specific inhibitors of ACSL4 and research on its upstream transcriptional regulators such as SP1 have shown clear cartilage-protective effects in OA models, suggesting that future strategies combining GPX4 modulation to enhance defense with ACSL4 inhibition to reduce attack may achieve more potent and synergistic therapeutic outcomes (He W. et al., 2023) (Figure 1).

### 3.5 Parallel defense pathways: GPX4, DHODH, and FSP1

Although the GPX4–GSH axis is widely recognized as the primary defense line against plasma membrane lipid peroxidation and ferroptosis, recent discoveries have identified GPX4-independent parallel defense mechanisms within cells, revealing the complexity and redundancy of the cellular antioxidant network. These parallel mechanisms primarily include mitochondrial DHODH and the plasma membrane-localized Ferroptosis Suppressor Protein 1 (FSP1)–CoQ10 system (Cao J. et al., 2025). DHODH is a key enzyme in the *de novo* pyrimidine nucleotide synthesis pathway, located in the inner mitochondrial membrane. Beyond its classical metabolic role, research has found that DHODH can limit lipid peroxidation within mitochondria by maintaining its own mitochondrial CoQ/CoQH_2_ redox pool, thereby independently suppressing ferroptosis (Mao et al., 2021; Deng et al., 2025). On the other hand, FSP1, initially considered a pro-apoptotic factor, has been newly established as a potent anti-ferroptotic protein. Localized to the plasma membrane, FSP1 utilizes NAD(P)H to reduce CoQ10 to CoQ10H_2_ or ubiquinol, which acts as a lipophilic radical-trapping antioxidant that can effectively interrupt the lipid peroxidation chain reaction, thus establishing a defense line on the plasma membrane independent of the GPX4–GSH system (Mao et al., 2021; Deng et al., 2025). These important findings suggest that under pathological conditions where GPX4 function is insufficient or inhibited, enhancing DHODH or FSP1 activity pharmacologically or genetically holds potential as an effective alternative or compensatory therapeutic strategy. Conversely, in fields like cancer therapy, inhibiting DHODH is being explored as a strategy to enhance ferroptosis in cancer cells and improve chemotherapy efficacy (Mishima et al., 2023). In OA research, data on the expression, function, and interaction of DHODH and FSP1 with GPX4 are gradually accumulating (Xia S. et al., 2025). In-depth investigation into the tissue-specific functions of these parallel pathways in chondrocytes and their crosstalk with the main GPX4 pathway holds significant translational medical importance for developing novel OA treatment strategies (Figure 1). Nevertheless, there is currently a lack of OA-specific genetic models validating the FSP1 or DHODH targets, as well as systematic research on the expression profiles and activity distribution of these pathways in different tissues such as cartilage, synovium, and subchondral bone. Therefore, at this stage, it cannot be asserted that they hold equal importance to GPX4 in OA. Furthermore, existing literature seldom discusses the synergistic or compensatory relationships between GPX4 and these parallel pathways. Whether they compensate for each other in OA or play dominant roles at different pathological stages remains an unknown area. Future systematic analysis of the tissue specificity and disease stage dependency of these pathways will directly impact the design of OA ferroptosis intervention strategies.

## 4 Therapeutic strategies targeting GPX4 for OA

### 4.1 Drugs directly Inhibiting lipid peroxidation or enhancing GPX4 activity

#### 4.1.1 Classical ferroptosis inhibitors

Ferrostatin-1(Fer-1) and Liproxstatin-1(Lip-1) are among the first identified and widely used specific small molecule inhibitors of ferroptosis. They have demonstrated potent inhibitory effects on the lipid peroxidation process in various cell models and animal disease models, including OA (Xu W. et al., 2023). In OA studies, direct intra-articular injection of Fer-1 into mouse or rat models significantly reduced the accumulation of lipid peroxidation end-products like MDA and 4-HNE in cartilage tissue, alleviated the degree of ECM degradation, and improved overall joint pathology scores. These results provide important proof-of-concept for treating OA by inhibiting ferroptosis (Wang X. et al., 2022; Pan et al., 2024). Another study showed that intra-articular injection of Botulinum Toxin A inhibited cartilage degradation in an OA model rat, and Lip-1 could further enhance this chondroprotective effect (Zeng et al., 2024). Related molecular mechanism studies using immunohistochemistry and Western blot analysis revealed significantly decreased GPX4 protein levels and upregulated pro-ferroptotic proteins ACSL4 and p53 in the OA cartilage layer; Lip-1 injection effectively reversed these changes in key protein expression (Cheng et al., 2024) (Table 1). Although these classical inhibitors show great potential in preclinical research, their clinical translation still faces substantial obstacles, including poor metabolic stability, suboptimal pharmacokinetics, and the critical fact that no anti-ferroptosis drug has yet entered human trials. Particularly noteworthy, systemic administration might interfere with normal redox balance in other tissues and organs. Therefore, localized administration methods, such as intra-articular injection or combination with sustained-release carriers, are considered more feasible and safer clinical translation pathways.

**TABLE 1:** Summary of agents targeting GPX4 for OA treatment.

Category	Compound/Intervention	GPX4 upstream/Downstream pathway or target	Action/Function	References
Classical ferroptosis inhibitors	Ferrostatin-1	ROS	Directly quenches lipid peroxyl radicals, stabilizes cell membrane structure, often used for comparison with other ferroptosis inhibitors	Chen B. Y. et al. (2024), Wang D. et al. (2023), He W. et al. (2023), Wang X. et al. (2022), Pan et al. (2024), Wipplinger et al. (2025), Zhu et al. (2024), Lv Z. et al. (2022)
	Liproxstatin-1	ROS	Inhibits lipid peroxidation. Often used for comparison with other ferroptosis inhibitors	Zeng et al. (2024), Xu Y. et al. (2023), Cheng et al. (2024)
Natural products/Small molecule agonists	Bushen Chushi Formula	MAPK/SLC7A11/GPX4 axis	Inhibits ferroptosis and inflammatory response, preserves cartilage structure, enhances subchondral bone quality	Li et al. (2025)
	Botulinum toxin A, Icariin, α-ketoglutarate	SLC7A11/GPX4 axis	Alleviates OA development by inhibiting chondrocyte ferroptosis	Luo and Zhang (2021), Xiao et al. (2024), Zeng et al. (2024), He Q. et al. (2024)
	Duhuo Jisheng Decoction, Vinpocetine, Protopine; et al.	Keap1/Nrf2/GPX4 axis	Inhibits ferroptosis, thereby attenuating cartilage degeneration, subchondral remodeling, synovitis, and ECM degradation	Wang Q. et al. (2024), Wu et al. (2025b), Chen H. et al. (2024), Tang et al. (2022)
	Amygdalin and magnesium ions	IL-17/GPX4 axis	Inhibits fibrocartilage formation and promotes hyaline cartilage regeneration	Zhao et al. (2025a)
	Qianghuo Shengshi decoction, Notopterol, Semaphorin 5A	PI3K/Akt/GPX4 axis	Prevents lipid peroxidation, inhibits ferroptosis, maintains cartilage integrity, thereby delaying OA progression	Zhuang et al. (2025), Zhou et al. (2025b), Cheng et al. (2022)
	β-Diketone Functionalized Microspheres, Ruscogenin, Biochanin A	Nrf2/SLC7A11/GPX4 axis	Alleviates chondrocyte lipid peroxidation, inhibits ferroptosis, slows OA progression	Ruan et al. (2024), He Q. et al. (2023), Xu H. et al. (2025)
	Paeonol, Quercetin	AMPK/Nrf2/GPX4 axis	Reduces lipid peroxidation damage, prevents inflammatory injury by inhibiting ferroptosis, improves articular cartilage destruction	Dong et al. (2025), Gong et al. (2025)
	Asperosaponin VI, bilirubin, Geniposidic acid	Nrf2/GPX4/HO-1 axis	Inhibits chondrocyte ferroptosis, improves OA.	Zhang Z. et al. (2025), Zhao et al. (2025b), Sun et al. (2024)
	Vitamin K2	MAPK/NFκB/Nrf2 axis	Reduces chondrocyte ferroptosis to slow down degradation of chondrocyte extracellular matrix	He R. et al. (2024)
	paeoniflorin	p53/SLC7A11/GPX4 axis	Inhibits ferroptosis, alleviates subchondral bone loss and cartilage destruction	Wu et al. (2025a)
	Urolithin A	AMPK/mTOR/HIF-1α axis	Inhibits inflammation and ferroptosis	Wang X. et al. (2025)
	Pantothenic acid, Kruppel-like factor 2, Sappanone A, Kukoamine A, Brevilin A	SIRT1/Nrf2 axis	Inhibits ferroptosis and improves mitochondrial dysfunction in chondrocytes, thereby improving OA.	Ruan et al. (2023), Liu et al. (2025), Shi et al. (2024), Zhang et al. (2024), Sun J. et al. (2023)
	SDF-1	IL6/HIF-1α axis	Increases MMP13 expression and secretion, leading to chondrocyte ferroptosis	Yang T. et al. (2025)
Regulating iron homeostasis & substrate supply	DFO	ROS	Chelates intracellular free iron ions, reduces ROS generated by Fenton reaction. Often used for comparison with other ferroptosis inhibitors	Miao et al. (2022), Gong et al. (2023)
	Selenoprotein	ROS	Indirectly enhances GPX4 defense capability	Xue et al. (2018)

#### 4.1.2 Natural products and small molecule agonists

Numerous recent literature reports that various natural products and synthetic small molecules can effectively inhibit chondrocyte ferroptosis by activating the SLC7A11/GPX4 axis or the Nrf2–GPX4 signaling pathway. For example, Icarin was confirmed to inhibit ECM degradation and ferroptosis in chondrocytes, thereby alleviating articular cartilage damage in OA rats by restoring SLC7A11 and GPX4 expression (Xiao et al., 2024). Quercetin was demonstrated to inhibit ferroptosis and improve joint cartilage destruction and degradation, delaying OA progression, by activating the AMP-activated protein kinase (AMPK)/Nrf2/GPX4 signaling pathway (Dong et al., 2025). Taurine showed potential to inhibit chondrocyte ferroptosis by upregulating OGT/GPX4 signaling, suggesting taurine supplementation as a potential therapeutic strategy for OA (Zhou et al., 2025a). Additionally, α-Ketoglutarate(α-KG) inhibited ferroptosis, significantly reducing matrix degradation and apoptosis in chondrocytes *via* the ETV4/SLC7A11/GPX4 signaling pathway, showing future clinical application prospects (He R. et al., 2024). Such natural compounds often offer advantages like lower toxicity, wide sources, and frequent multi-target synergistic effects. However, they commonly face challenges such as low bioavailability, complex action targets, difficulty achieving effective therapeutic concentrations in target tissues, and rapid *in vivo* metabolism. In clinical advancement, strategies with more realistic value include local delivery or use as adjuvant therapy, rather than relying on long-term systemic exposure. Current research hotspots involve developing derivatives with improved pharmacokinetic properties based on these lead compounds, or utilizing advanced drug delivery systems like nanoparticles, hydrogenated coconut oil microspheres, and degradable hydrogels to enhance their targeted delivery efficiency and therapeutic index (Table 1).

#### 4.1.3 Indirect protection of GPX4 by regulating iron homeostasis and substrate supply

Beyond directly targeting GPX4 or its related pathways, indirect strategies have also garnered attention, primarily including reducing local iron load for example, using iron chelators (Ghaith et al., 2022; Wu et al., 2023), decreasing oxidizable PUFAs substrates in membrane phospholipids through dietary intervention or locally altering lipid composition (Li Y. et al., 2023), and supplementing cofactors essential for GPX4 enzymatic activity, such as selenium (Ingold et al., 2018; Jin Jung et al., 2023). These methods aim to indirectly protect chondrocytes by reducing upstream inducers of ferroptosis or enhancing GPX4’s defensive capacity. While iron chelators can reduce iron load-related injury in animal models, in clinical application, attention must be paid to potential systemic side effects of long-term systemic iron chelation therapy, such as anemia, immune disorders, and metabolic abnormalities. Nutritional interventions, such as appropriate selenium supplementation or antioxidant diets, can serve as long-term, adjuvant conditioning strategies. Several animal experiments suggest that local use of iron chelators or dietary selenium supplementation can alleviate OA progression, but these preliminary conclusions need further validation in more rigorous and standardized preclinical studies and subsequent clinical trials (Table 1). Overall, indirect regulation strategies are more suitable as adjuvant treatments, and their true clinical value still requires validation through population studies.

### 4.2 Gene therapy and RNA Intervention to elevate GPX4 or Inhibit promoters

#### 4.2.1 Direct delivery of GPX4 gene or upstream positive regulators

Gene therapy offers the potential for sustained upregulation of GPX4 levels. Local injection of adeno-associated virus (AAV) vectors or various nanocarriers into the joint cavity to deliver the GPX4 gene has been confirmed in animal models to achieve long-term, stable expression of this gene and show potential for improving cartilage morphology and function (Pang et al., 2024; Wang S. et al., 2025; Sheng et al., 2025; Yu et al., 2024). Using similar strategies for upstream positive regulators of GPX4, for example, upregulating SLC7A11 expression *via* gene therapy, can multi-targetedly enhance the cell’s overall antioxidant capacity and GSH supply, thereby indirectly supporting and enhancing GPX4 activity (Liao et al., 2024). More interestingly, research has found that using siRNA to knock down the Growth factor independence 1 (Gfi1) gene in chondrocytes, or overexpressing this gene *via* intra-articular AAV-Gfi1 injection, inactivated the MAPK signaling pathway, subsequently observing downregulation of ferroptosis promoters such as Cox2 and Acsl4 and upregulation of ferroptosis inhibitors such as GPX4 and SLC7A11, ultimately alleviating cartilage degeneration, osteophyte formation, and synovitis, and improving OA progression (Jin et al., 2025). Although promising, gene therapy still faces key challenges, including vector immunogenicity, achieving precise tissue-specific expression regulation, safety assessment of long-term expression, and controllability of expression intensity (Jakabek et al., 2025). Even with local injection, the clearance and response of the synovial and joint cavity immune microenvironment to the vector must be strictly assessed. For OA, employing local intra-articular administration is expected to significantly reduce systemic exposure and related immune reactions, thus being considered a more realistic clinical translation pathway (Lin et al., 2024; De la Vega et al., 2025) (Table 2).

**TABLE 2:** Statistics of gene therapy and RNA interventions to elevate GPX4 or inhibit promoters for OA treatment.

Category	Compound/Intervention	GPX4 upstream/Downstream pathway or target	Action/Function	References
Direct delivery of GPX4 gene or upstream positive regulators	Adeno-associated virus-overexpressing *Gfi1*	MAPK	Alleviates cartilage degeneration, subchondral bone sclerosis and synovitis	Jin et al. (2025)
	pcDNA3.1-NF-κB p65 expression plasmid	NF-κB	Reduces ferroptosis in chondrocytes	Chen et al. (2025)
	Adenovirus expressing KLF2	SIRT1	Improves mitochondrial dysfunction in chondrocytes, thereby improving OA.	Shi et al. (2024)
	GPX4 overexpression plasmids	ROS	Reduces chondrocyte apoptosis, alleviates joint symptoms	Pang et al. (2024)
	Short hairpin RNA of FBXW7	SLC7A11	Reduces chondrocyte damage, improves OA cartilage injury	Yang et al. (2026)
miRNA/lncRNA	miRNA-19b-3p	SLC7A11	Enhances chondrocyte ferroptosis	Kong et al. (2023)
	miR-181a-5p	SECISBP2	Enhances chondrocyte ferroptosis	Xue et al. (2018)
	LncRNA Meg3	SLC7A11	Reduces chondrocyte ferroptosis	Zhu et al. (2024)
Histone modifications and DNA methylation	P21	GPX4 ubiquitination	Inhibits GPX4 ubiquitination, reduces chondrocyte ferroptosis, improves OA cartilage injury	Zheng et al. (2024)
	METTL14	GPX4 methylation	Inhibits GPX4 methylation, alleviates chondrocyte degeneration, oxidative stress and ferroptosis	Liu M. et al. (2024)
	Gossypol Acetic Acid	GPX4 methylation	Inhibits GPX4 methylation, alleviates ferroptosis in OA chondrocytes	Shang et al. (2025)
	4-octyl Itaconate	GPX4 methylation	Inhibits GPX4 methylation, reduces chondrocyte degeneration, oxidative stress and ferroptosis	Pan et al. (2024)

#### 4.2.2 miRNA/lncRNA regulation of GPX4 gene

Numerous studies indicate that non-coding RNAs, particularly miRNAs and lncRNAs, play key roles in the fine-tuning regulation of GPX4 and its upstream/downstream network (Zhao Y. P. et al., 2025; Liu M. et al., 2024). Specifically, certain miRNAs can target and regulate iron metabolism-related proteins or directly inhibit the expression of System Xc^−^ and GPX4, thereby promoting ferroptosis (Fan et al., 2021; Xu et al., 2021). For instance, in an IL-1β-stimulated chondrocyte OA model and a surgically induced OA rat model, exosomes derived from OA patient FLS were found to reduce chondrocyte viability, GSH/GSSG ratio, GPX4, SLC7A11, and MMP levels; the miR-19b-3p mimic carried by these exosomes could further enhance this effect, exacerbating cartilage ferroptosis and damage (Kong et al., 2023). Therefore, using miRNA inhibitors or locally delivering miRNA mimics has become an active direction in current intervention research. On the other hand, lncRNAs have also been confirmed to participate in regulation. Research indicates that specific lncRNAs can interact directly with the p53 gene and trigger ferroptosis *via* the p53–GPX4 axis (Chen C. et al., 2021). For example, silencing the lncRNA MEG3 in chondrocytes increased miR-885-5p expression while downregulating SLC7A11 and GPX4, thereby making chondrocytes more sensitive to ferroptosis (Zhu et al., 2024). However, challenges related to *in vivo* delivery efficiency, off-target effects, and cell specificity remain key bottlenecks for RNA intervention. Future studies should systematically investigate long-term toxicity, immune responses, and distribution in cartilage and synovial tissues. Effective biomarkers, such as GPX4, 4-HNE, and MDA in joint fluid, can be used to monitor treatment response and assist in dose optimization (Table 2).

#### 4.2.3 Histone modifications and DNA methylation

Gene expression activity is profoundly influenced by chromatin structure and epigenetic modifications. Among these, histone modifications like ubiquitination and DNA methylation are important mechanisms regulating gene transcriptional status. In a DMM-induced mouse OA model, research found that the p21 protein significantly affected the recruitment of GPX4 to the linear ubiquitin chain assembly complex(LUBAC) and regulated the M1-linear ubiquitination level of GPX4. The absence of p21 reduced GPX4 protein stability *in vivo* and exacerbated cartilage degradation, indicating that p21 plays an important anti-ferroptotic role in OA by regulating GPX4 stability (Zheng et al., 2024). DNA methylation also plays a crucial role in the ferroptosis process, as it can regulate PUFA synthesis and ROS levels (Lee et al., 2020). On one hand, DNA methylation can suppress the activity of certain genes related to lipid peroxidation, thereby preventing ferroptosis; on the other hand, aberrant DNA methylation may lead to the silencing of pro-ferroptosis genes such as the GPX4 promoter region, thereby inducing ferroptosis (Zhang et al., 2022). In a monosodium iodoacetate-induced OA rat model, Methyltransferase-like 14 (METTL14) was confirmed to inhibit ferroptosis and ECM degradation in damaged chondrocytes by inhibiting m6A modification of GPX4 mRNA, a common RNA methylation, thereby protecting cartilage (Liu D. et al., 2024). Additionally, Gossypol Acetic Acid was reported to alleviate ferroptosis in OA chondrocytes by inhibiting GPX4 methylation (Shang et al., 2025). 4-Octyl Itaconate also showed effects in reducing chondrocyte degeneration, oxidative stress, and ferroptosis by inhibiting GPX4 methylation (Pan et al., 2024). These studies reveal the complex role of epigenetic regulation in OA ferroptosis and provide new potential intervention targets (Table 2).

### 4.3 Combination therapies and novel drug delivery systems

Given the multifactorial nature and complexity of OA pathogenesis, combination therapy strategies are generally considered to have greater advantages and potential than single-target interventions. Currently explored potential combination schemes mainly include the following aspects. First, combining locally applied anti-inflammatory drugs with ferroptosis inhibitors. Current first-line clinical treatments for OA include oral medications and intra-articular injections, with oral medications primarily being non-steroidal anti-inflammatory drugs (NSAIDs) like ibuprofen, aspirin, and celecoxib. Interestingly, in ferroptosis research, ibuprofen has been shown to downregulate the Nrf2 signaling pathway, thereby inducing ferroptosis in glioblastoma cells (Gao et al., 2020); aspirin was found to induce ferroptosis in a hepatocellular carcinoma cell model (Wang Y. F. et al., 2023); and celecoxib was demonstrated to alleviate ulcerative colitis in mice by inhibiting ferroptosis, suggesting it might also have the potential to modulate the ferroptosis process in OA (Li et al., 2024). Therefore, these NSAIDs, not only due to their existing anti-inflammatory effects but also their potential ferroptosis-regulating capabilities, might become promising candidates in OA combination therapy. In the future, combining classic ferroptosis inhibitors such as Fer-1 and Lip-1 or emerging drugs with traditional anti-inflammatory drugs to achieve dual protection by simultaneously inhibiting inflammation and lipid peroxidation might be a new strategy to effectively delay OA progression. Second, combining physical correction such as reducing joint load *via* braces or rehabilitation training with molecular intervention such as local injection of GPX4 activators or Fer-1-like drugs. For OA patients with mechanical overload, correcting the abnormal mechanical environment while performing targeted molecular intervention is expected to more significantly slow cartilage degeneration (Zhang Y. et al., 2025; Du et al., 2025; Han et al., 2024). Third, combining stem cell-derived exosomes or tissue engineering scaffolds with GPX4 enhancers. Using MSC exosomes or their modified products, or bioactive tissue engineering scaffolds, in combination with GPX4-enhancing small molecules, can promote tissue repair and regeneration while effectively reducing ferroptosis levels in implanted cells or host chondrocytes, thereby improving the survival rate and tissue integration of implants (Singhmar et al., 2025; Xia X. et al., 2025; Yang X. H. et al., 2025; Buford, 2025). The key to successfully implementing these combination strategies lies in several aspects: determining the optimal timing of administration such as initiating intervention at early or mid-stage OA, developing local delivery carriers capable of controlled drug release and properly managing their potential side effects, and ensuring good pharmacokinetic and pharmacodynamic compatibility between the combined drugs. Currently, some prospective animal studies have begun exploring schemes combining drug administration with advanced biomaterials, yielding positive preliminary results, but their long-term efficacy and safety still require evaluation in larger-scale, in-depth studies.

### 4.4 Practical considerations in pharmacokinetics and administration routes

The synovial cavity, as a natural enclosed space, provides an ideal window for local drug delivery, allowing drugs to act on the lesion site at high concentrations while minimizing systemic exposure and associated potential risks (Gerwin et al., 2006). Local injection, meaning intra-articular injection, has extensive clinical application experience for example, injections of hyaluronic acid or corticosteroids, and this mature administration route is equally suitable for delivering ferroptosis inhibitors or GPX4-related gene and RNA therapy products. However, conventional single intra-articular injections often have a short duration of action, as the drug is rapidly cleared or metabolized by the synovial tissue. Therefore, developing drug delivery carriers capable of sustained, controlled release, such as biodegradable microspheres, thermosensitive or pH-sensitive hydrogels, and various nanoparticles, is crucial for achieving long-term joint protection (Yu et al., 2024; Edwards, 2011). Furthermore, achieving selective drug delivery to chondrocytes or synovial cells by modifying the carrier surface with specific ligands such as antibodies or peptides targeting specific antigens on chondrocyte surfaces is expected to significantly improve therapeutic efficacy while further reducing the risk of side effects on other normal cells within the joint (Ferrari et al., 2015). Developing such efficient and precise targeted delivery systems is one of the key technological bottlenecks that must be addressed to successfully advance these innovative therapies towards clinical translation (Kavanaugh et al., 2016).

## 5 Discussion and perspectives

In recent years, in OA, a complex degenerative joint disease, increasing research indicates that ferroptosis not only exists in the pathological process but is also closely related to pathological features such as articular cartilage degeneration, synovial inflammation, and subchondral bone changes. Among the known ferroptosis regulatory pathways, the GPX4-dependent axis has been most extensively studied and identified as central to ferroptosis control. Due to its unique biochemical function of directly reducing lipid peroxides and blocking the oxidative chain reaction on cell membranes, GPX4 is often regarded as the central barrier or gatekeeping enzyme in the ferroptosis response. A large number of clinical samples and animal experiments show that GPX4 levels are significantly decreased in degenerative cartilage tissue, and the degree of its downregulation correlates with the severity of articular cartilage destruction, matrix degradation, and inflammation (Miao et al., 2022; Lv Z. et al., 2022; Ma et al., 2024). *In vitro* and *in vivo* models, knockout or inhibition of GPX4 can rapidly induce severe lipid peroxidation, mitochondrial rupture, and typical ferroptosis phenotypes in chondrocytes, further reinforcing its core role in maintaining joint homeostasis (Sun S. et al., 2023; Lu et al., 2024). Precisely because of this, targeting GPX4 and ferroptosis is gradually becoming a potential new therapeutic strategy in the OA field.

However, although GPX4 holds an irreplaceable executive position in ferroptosis regulation, viewing it alone as the optimal therapeutic target for OA is still insufficient. The occurrence of ferroptosis depends on multiple parallel and coupled molecular pathways, and the role of GPX4 within them is strictly constrained by upstream reductive systems, lipid substrate sources, and alternative antioxidant networks. First, as a GSH-dependent peroxide reductase, GPX4 activity highly depends on cystine provided by System Xc^−^/SLC7A11 to maintain GSH synthesis. Multiple studies have confirmed that SLC7A11 often shows significant downregulation under inflammatory stimulation, oxidative stress, or iron load conditions. Its dysfunction leads to GSH depletion, rendering GPX4 unable to effectively exert its antioxidant function even with adequate expression (Wu et al., 2025a; Wang D. et al., 2023). Second, ACSL4 and LPCAT3, as key lipid metabolism enzymes, determine the content and distribution of polyunsaturated fatty acids in membrane phospholipids, affecting cellular susceptibility to ferroptosis at the source. ACSL4 is generally upregulated in OA cartilage tissue, and its continuous activation expands the pool of oxidizable lipid substrates, thereby significantly increasing the tendency for ferroptosis (Guan et al., 2025; Zhou et al., 2023; Habaxi et al., 2024). This makes simply enhancing GPX4 difficult to fully counteract the pro-death signals from the substrate side. Furthermore, recent research reveals that cells possess multiple GPX4-independent anti-ferroptosis mechanisms. Among them, the membrane surface FSP1–CoQ10 system can effectively block the lipid radical chain reaction by maintaining ubiquinol or CoQ10H_2_ levels in the lipid membrane region, while mitochondrial inner membrane DHODH exerts local antioxidant effects by regulating the redox state of mitochondrial CoQ/CoQH_2_ (Mao et al., 2021). These pathways can provide important compensation when GPX4 is partially impaired, also indicating that ferroptosis regulation has high redundancy, and single-target intervention may not completely block the pathological process. From a therapeutic perspective, the central role of GPX4 is not equivalent to the only critical node. Its efficacy is limited by substrate supply, lipid composition, the status of parallel protective pathways, and factors such as inflammation, mechanical load, and aging present in the joint microenvironment. Based on this understanding, future research should conduct deeper analysis of the joint-specific regulatory network of ferroptosis at a systems level. For example, simultaneously evaluating the expression, enzyme activity, and lipidomic characteristics of GPX4, SLC7A11, ACSL4, LPCAT3, FSP1, and DHODH in the same batch of patient tissues, combined with disease staging and tissue-specific differences between cartilage and synovium, can clarify the dominance of each pathway at different stages. More importantly, it is necessary to use conditional gene knockout, overexpression models, and cross-validation with various OA models to distinguish the causal sequence, compensatory responses, and synergistic relationships between different molecules, laying the foundation for establishing truly precise and effective intervention strategies.

However, current research still faces many challenges that hinder the clinical translation of ferroptosis targeting strategies. First, the regulatory network of ferroptosis is extremely complex, involving interactions among iron metabolism, lipid chemistry, ROS generation and scavenging systems, and multiple signaling pathways. GPX4, together with other ferroptosis inhibitory pathways such as FSP1/CoQ10 and BH4/DHFR axes, maintains the cellular antioxidant state. Their functional redundancy and interactivity mean the specific contribution of a single intervention strategy under different cell types and joint microenvironment conditions is not entirely clear. Most current OA animal models, such as the DMM model or chemically induced models, can simulate cartilage damage in the short term but struggle to fully replicate the long-term cumulative multifactorial pathological changes of human chronic OA. These models may have significant biases when evaluating the long-term efficacy and safety of GPX4 activation or ferroptosis inhibition strategies. The physiological differences between models and human disease constitute a significant obstacle in current research, also complicating the interpretation of cross-model comparison results.

Clinically, a prominent and unresolved challenge is the lack of sensitive, specific, and easily accessible biomarkers to accurately and non-invasively detect intra-articular ferroptosis activity or GPX4 functional status. Although there is exploration of indirectly reflecting ferroptosis activity by detecting lipid peroxidation products such as MDA and 4-HNE, iron metabolism indicators, or GPX4 expression in joint fluid (Xia S. et al., 2025), these indicators are still in the exploratory stage, with no standardized detection protocols established or their clinical significance validated in large-scale clinical samples. Furthermore, levels of lipid peroxidation products are often influenced by other inflammatory responses and metabolic states, lacking specificity. Promoting the development of clinically detectable early biomarker tools is particularly important. An ideal strategy should integrate imaging such as quantitative MRI parameters, fluid aspiration measuring MDA, 4-HNE, iron metabolism indicators, and GPX4-related protein/nucleic acid levels, and advanced omics analyses to build a comprehensive risk assessment system for predicting ferroptosis activity and treatment response (Lotz et al., 2013). This is of great significance for improving enrollment criteria in clinical trials, quantifying efficacy indicators, and achieving treatment personalization.

Individual patient differences such as age, metabolic status such as diabetes, obesity, chronic inflammation levels, and medication history can significantly impact the GPX4/GSH antioxidant axis and iron homeostasis balance. These differences may lead to inconsistent performance of the ferroptosis regulatory network in different subjects, thereby interfering with the efficacy of GPX4-targeted therapy. This requires finer stratification of subjects in clinical trial design, conducting subgroup analysis based on clinical phenotypes, metabolic status, and biomarker levels to screen patient populations most likely to benefit. Additionally, since ferroptosis intersects with various normal physiological signals such as ROS signaling, systemic long-term inhibition of ferroptosis may interfere with normal cellular functions or even weaken the body’s natural defense response against pathologies like tumors (Xu H. et al., 2025). This double-edged effect means relying solely on systemic GPX4 activation or broad-spectrum ferroptosis inhibitors is not an ideal strategy. Therefore, developing local, controllable, and reversible therapeutic methods holds greater clinical advantages, but this largely depends on the development of more advanced delivery systems and precise monitoring protocols.

The main lesion site in OA is concentrated in the articular cartilage region, and its early key pathological changes are highly correlated with cartilage degeneration. However, OA is not a simple cartilage degenerative disease but a complex condition involving multiple tissues and cells, including cartilage, synovium, subchondral bone, osteophyte formation, joint capsule, and local immune cell populations. Current experimental systems related to GPX4 research mostly focus on chondrocytes, with clearly insufficient research evidence in synovium, immune cells, and even subchondral bone cells. Therefore, the analysis of GPX4 should proceed from a more systemic and holistic joint structure perspective. To deepen the understanding of ferroptosis in OA, future research must shift from a single-target, single-cell perspective to a multi-cell type, cross-tissue integration perspective. Combining cutting-edge technologies such as single-cell omics, spatial transcriptomics, metabolomics, and tissue-specific knockout models for joint analysis of different disease stages and tissues within the joint will reveal the differential regulation of GPX4 in different cell populations.

In the field of delivery technology, targeting the unique joint anatomy and pathological environment of OA, developing biodegradable sustained-release carriers such as smart responsive nanogels, porous microspheres, or tissue engineering scaffolds is key to achieving long-term and controlled release of GPX4 genes, regulatory miRNAs or ASOs, or small molecule ferroptosis inhibitors at the lesion site (Wang Y. et al., 2022). Modifying the carrier surface with targeting molecules such as aptamers or functional peptides can improve local drug retention and specificity in diseased cartilage or pathological synovial cells, while reducing potential adverse effects from systemic exposure.

Considering the multifactorial pathogenesis of OA, future research needs to prioritize conducting early proof-of-concept clinical trials to evaluate the feasibility and synergistic effects of locally applied ferroptosis inhibitors such as Fer-1 or GPX4 activators combined with existing conservative treatments including weight management, physical therapy, and NSAIDs or emerging cell and tissue engineering therapies. Such studies need to comprehensively integrate objective structural improvement indicators through quantitative MRI or arthroscopic evaluation and subjective clinical outcome measures such as pain and function scores, while focusing on treatment safety, local and systemic pharmacokinetics, and dynamic changes in preset biomarkers.

Achieving these long-term goals relies on deepening interdisciplinary collaboration, including close cooperation among basic mechanism researchers, biomaterial scientists, clinical orthopedics and rheumatology experts, and pharmacokinetics and toxicology assessment experts. Establishing unified and standardized OA animal models, local administration protocols, and comprehensive efficacy evaluation systems will be key to promoting the translation of ferroptosis-targeted therapies from the laboratory to the clinic. Only through such full-chain, multi-domain collaborative efforts can current challenges be systematically overcome, accelerating the clinical application of novel OA treatment strategies based on GPX4 and ferroptosis regulation.

## 6 Conclusion

In summary, increasing research evidence supports a key role for ferroptosis in the occurrence and progression of OA. Within this context, GPX4 is considered an important core regulatory factor connecting redox imbalance, lipid peroxidation, and joint tissue degeneration. A growing body of preclinical research suggests that targeting the ferroptosis regulatory pathway centered on GPX4, including drug inhibitors, natural active compounds, gene or RNA intervention strategies, and novel local delivery systems, can alleviate cartilage degeneration and joint inflammation to varying degrees in experimental OA models. However, ferroptosis in OA is not dominated by a single molecular node but relies on multiple parallel and coupled molecular pathways. GPX4 is strictly constrained by upstream reductive systems, lipid substrate sources, and alternative antioxidant networks. These pathways can provide important compensation when GPX4 is partially impaired. Before advancing these strategies to clinical application, several key challenges must be addressed, such as the complexity and functional redundancy of the ferroptosis regulatory network, the limitations of existing animal models in simulating the pathological features of human chronic OA, and the lack of reliable biomarkers for assessing intra-articular ferroptosis activity in humans. Future research should adopt integrated, staged, and tissue-specific research strategies to systematically elucidate the precise modes of action of GPX4 and ferroptosis in different joint tissues and disease stages. Efforts should be made to promote a shift from merely alleviating symptoms to truly disease-modifying treatment strategies that protect joint structure and function, thereby providing a new theoretical basis and translational direction for precise intervention in OA.
